# Magnitude and factors associated with institutional delivery service utilization among childbearing mothers in Cheha district, Gurage zone, SNNPR, Ethiopia: a community based cross sectional study

**DOI:** 10.1186/s12884-015-0716-8

**Published:** 2015-11-17

**Authors:** Feleke Habte, Meaza Demissie

**Affiliations:** Johns Hopkins University – Technical Support for the Ethiopian HIV/AIDS ART Initiative project, Addis Ababa, Ethiopia; Addis Continental Institute of Public Health, Addis Ababa, Ethiopia

**Keywords:** Institutional delivery, Skilled attendant, Cheha district, Ethiopia

## Abstract

**Background:**

Ethiopia is one of the six countries that contributes’ to more than 50 % of worldwide maternal deaths. While it is revealed that delivery attended by skilled provider at health facility reduced maternal deaths, more than half of all births in Ethiopia takes place at home. According to EDHS 2011 report nine women in every ten deliver at home in Ethiopia. The situation is much worse in southern region. The aim of our study is to measure the prevalence and to identify factors associated with institutional delivery service utilization among childbearing mothers in Cheha District, SNNPR, Ethiopia.

**Methods:**

A community based cross sectional survey was conducted in Cheha District from Dec 22, 2012 to Jan 11, 2013. Multistage sampling method was employed and 816 women who gave birth within the past 2 years and lived in Cheha district for minimum of one year prior to the survey were involved in the study. Data was entered and analyzed using Epi Info Version 7 and SPSS Version 16. Frequencies and binary logistic regression were done. Factors affecting institutional delivery were determined using multivariate logistic regression.

**Results:**

A total of 31 % of women gave birth to their last child at health facility. Place of residence, ability to afford for the whole process to get delivery service at health facility, traveling time that takes to reach to health institution which provides delivery service, husband’s attitude towards institutional delivery, counseling about where to deliver during ANC visit and place of birth of the 2^nd^ youngest child were found to have statistically significant association with institutional delivery.

**Conclusion:**

Institutional delivery is low in the study area. Access to health service was found to be the most important predictor of institutional delivery among others. Accessing health facility within reasonable travel time; providing health education and BCC services to husbands and the community at large on importance of using health institution for delivery service; working to improve women’s economic status; counseling women to give birth at health institution during their ANC visit and exploring the overall quality of ANC service are some of the areas where much work is needed to improve institutional delivery.

## Background

The inter-agency (WHO, UNICEF, UNFPA and the World Bank) report on maternal mortality shows that the ratio of 99 % of deaths in developing countries and only 1 % of deaths in developed countries has not been changed since 1986 although maternal deaths in 2010 have declined to 287,000 from over 500, 000 death report in 1986 by WHO [1, 2]. Even if the maternal mortality has fallen considerably worldwide, the overall aim of MDG 5 (a 75 % reduction) is very unlikely to be achieved by 2015, unless there are remarkable further reductions in the remaining years.

It is a well established fact that most maternal deaths occur due to complications that arise during labor, delivery and the immediate postpartum period, with obstetric hemorrhage being the main medical cause of maternal death [3]. Therefore, prevention of maternal mortality requires prioritization of the intrapartum period, and based on the available empirical evidence a health centre intrapartum-care strategy can be justified as the best bet to bring down high rates of maternal mortality [4]. The risk of maternal deaths and disabilities outside the intrapartum period can be reduced through other appropriate reproductive health services such as antenatal care (ANC), postnatal care (PNC), family planning and safe abortion [4, 5]. The safe motherhood initiative also strongly emphasizes ensuring the availability and accessibility of skilled care at the time of childbirth, of which institutional delivery is one element where emergency obstetric care could be found to handle complicated cases. This is critical intervention to avoid most maternal deaths occurring from preventable obstetric complications [6].

It has been also estimated that the presence of skilled attendants at delivery could reduce maternal deaths by 16 to 33 % [7] and it was also reported that around 20–30 % of neonatal mortality could be reduced by implementing skilled birth care services [8]. Skilled attendance is the process by which a woman is provided with adequate care during labour, delivery and the early postpartum period. The process requires a skilled attendant AND an enabling environment which includes adequate supplies, equipment and infrastructure as well as efficient and effective systems of communication and referral [7]. Safe delivery ensures that all deliveries are attended by persons with the right knowledge, skills and equipment and also provide post-partum care to mother and baby [9]. In addition to professional attention, it is important that mothers deliver their babies in an appropriate setting, where life saving equipment and hygienic conditions can also help reduce the risk of complications that may cause death or illness to mother and child [4, 10]. Hence implementation of an effective intrapartum-care strategy is an overwhelming priority for decreasing maternal mortality [4]. In this study we used institutional delivery attended by skilled birth attendants to measure the safest delivery service utilization among childbearing mothers at birth owing to the extreme rareness of unskilled attendance of delivery at a health facility which is labeled to give delivery service in Ethiopia, and also on account of the extreme rareness of skilled birth attendance at home in the country - and if at all the delivery is attended by skilled providers at home it is difficult to decide whether this delivery service is actually to be safe according to the above definitions.

Despite the fact that a health centre intrapartum-care strategy can be justified as the best bet to bring down high rates of maternal mortality [4], more than half of births in developing countries are reported to take place at home [11]. Likewise, although skilled birth attendance could also reduce maternal deaths, more than half of all births in Sub-Saharan Africa still take place without the assistance of skilled birth attendants and far from emergency services. The lowest level of skilled birth attendance (33.7 %) occur in Eastern Africa (where Ethiopia resides) as opposed to more than 99 % coverage in developed regions [12, 13].

Various studies from many parts of the world identified factors that lead to low utilization of health facilities for delivery service. It includes factors related to place of residence and socioeconomic status such as women’s age, ethnicity, education, religion, culture, clinical need for care and decision-making power. The costs, location, quality and “demand factors” of health services were also found to be important [14]. There is a 5-fold higher rate of skilled attendance at birth among the least poor versus the most poor in most countries [15]. Distance has been established as one of the major barriers to health care [16, 17]. Long distance can be an impediment to reaching a health facility as well as a deterrent to even try to seek care. A substantial proportion of maternal deaths in developing countries occur on the way to hospital; other women are almost beyond help by the time they arrive [3, 18].

A research done in different parts of Ethiopia, found that residential area, parity, antenatal care utilization, maternal education, husband’s education, knowledge of mothers on pregnancy and delivery services, husbands and mothers attitude towards institutional delivery, women’s decision making power, women’s age at interview less than 20 years, monthly income and history of intrapartum complications were most significant predictors of institutional delivery service utilization [19–22]. A study done in southern Ethiopia found that terrible health institutional delivery experience limited women’s ability to seek care for subsequent pregnancies [23]. Another study done in Ethiopia found that the pregnant woman was influenced by her attendants for seeking care at health facilities; families only seek care for complications if local or herbal, remedies and prayer were defeated. Timely care seeking was reliant on the knowledge, understanding and financial means of the husband. Distance, cost and lack of support for the cultural practices around birth were also hindering factors for childbearing mothers to give their birth at health facility [24].

The 2011 Ethiopian Demographic and Health Survey (EDHS) reported MMR of 676/100,000 live births which is not significantly different from those reported on 2000 and 2005 which were 871 and 673 per 100,000 live births respectively with overlapping intervals indicating the difficulty in achieving the 2015 target of maternal mortality ratio (MMR) for Ethiopia which is 218/100,000 live births [25]. Although it was indicated that a health centre intrapartum-care strategy can be justified as the best bet to bring down high rates of maternal mortality, the proportion of births attended at health facility in Ethiopia is still very low, which is only 9.9 %, and this figure is even worse (6.2 %) in Southern Region where Cheha district is located [25].

To curb the burden of high maternal mortality in Ethiopia, the low institutional delivery (attended by skilled attendants) service coverage has to be improved. This urges the need to elucidate factors that affect institutional delivery service utilization. Therefore, this study aimed at measuring the magnitude and identifying the factors that influences institutional delivery service utilization in Cheha district, Ethiopia. The identified factors can be an input for policy makers, planners and health managers so that the government of Ethiopia can take appropriate actions on these factors to enhance institutional delivery service utilization- which can in turn contributes for a considerable reduction of maternal mortality in the district in particular and in Ethiopia in general.

## Methods

### Study area

Cheha District is located in Gurage Zone, Southern Nations Nationalities and Peoples Region (SNNPR)-Ethiopia. Emdebir town the capital city of Cheha District is located 182 Km south west to Addis Ababa. The district has 2 small towns, of which one is the capital city, and 39 rural “kebeles” (smallest administrative units). It has a population of 138,054 out of which 67,094 of them are male and 70, 960 are female. Out of the total population childbearing mothers comprises 23.3 % (32,167). Most people are economically dependent on Agriculture. There is 1 hospital (owned by Faith Based Organization), 6 health centers, 7 clinics and 38 health posts in the district. Initial assessment of health institutions was done in collaboration with the District Health Office; then those institutions that can actually provide safe delivery service, out of the aforementioned health institutions, were identified.

### Study design, period, population and sampling procedures

Community based cross sectional survey was conducted in Cheha District from December 22, 2012 to January 11, 2013. The source populations were all women of childbearing age (15–49 years) in Cheha District who had experience of at least one birth. The actual study populations were a randomly selected women who had given birth in the past 2 years in Cheha District prior to the survey and who lived at least 1 year in the district prior to the survey. Women who were mentally or physically ill and not capable to be interviewed were excluded from the study. The sample size was calculated using Epi Info version 7. A sample size of 845 was calculated using formula for single population proportion, taking the largest p value, prevalence (*P*) of delivery attended by skilled personnel which is 10 % for the country, assuming a design effect of 2, a margin of error of 3 % at 95 % confidence interval, and a non-response rate of 10 %.

Multistage sampling method with stratification of the district into rural and urban areas was used. From the district’s 39 rural kebeles, 8 rural kebeles were selected randomly and from the two urban areas having 3 kebeles, 2 kebeles’ were randomly selected and included in the study. In the selected kebeles, households having the target were identified by house to house visit (i.e. by census) using 30 health extension workers (high school graduates who undergo one year training program to deliver mainly packages of preventive and health promotion services and few basic curative services). In the census women who gave birth within the past 2 years in Cheha district, and lived a minimum of 1 year in the district, prior to the survey were identified and this was used to randomly select the study subjects. Then sample size was allocated to each urban and rural area proportional to size of the target households, and simple random sampling was applied to get the required households from households having the target. Whenever two or more eligible women were found in the same household only one of them was selected randomly and included in the study.

### Data collection procedures

Interviewer administered pre-tested structured questionnaire which was used in previous studies and adapted according to the facts obtained from literature review was employed to collect data. The questionnaire was translated to local languages (Amharic and Guragigna) to facilitate the interview.

The data was collected by female nurses who can read and fluently speak both Amharic and Guragigna languages after providing them with a three days of training on objective of the study, techniques of survey interviewing using the questionnaire and on the overall data collection procedures by the principal investigator. Care was taken not to assign the nurse in the catchment kebele of the health institution where they work. In addition, six male supervisors (BSc nurses and BSc in Public Health) were trained on supervision techniques in addition to training on data collection procedures. The training was complemented by practical session. Moreover, Pre - testing of the questionnaire had been conducted in one rural kebele of Eza district which is neighbor to Cheha district, before the actual data collection date, and accordingly correction, such us adjustment of skip points, improvement in translation to local lanquage and other improvements had been made on the final version of the questionnaire. The data was collected in mothers own home at their convenient time. A strong supervision of the data collection process had been carried out by principal investigator and all supervisors.

### Data management and analysis

Information were pre coded, the data was checked for errors, and missing values had been dealt with. Categorization was made for those data that were not pre-categorized. Coded data was entered using Epi Info version 7 and was exported to SPSS for Windows version 16 for analysis. Data cleaning, recoding and verification were done accordingly. Frequency and measure of variations were used to describe the study population. Bivariate analysis using logistic regression technique was done to see the association of each independent variables with the outcome variable and crude odds ratio with 95 % CI were computed. Those variables which had a significant association (with *p* value of less than 0.05) with the outcome variable including the well known confounders were included in multivariate logistic regression model. In doing multivariate logistic regression each independent variables having *p* value of less than 0.05 in bivariate analysis and the well known confounders were grouped (Group of independent variables include: Socio-demographic variables including access related factors, health care behaviours and cultural factors; obstetric variables including characteristics of ANC service; knowledge and attitude of respondents) and each of these group of independent variables were entered in different blocks in SPSS covariate box and we ran SPSS to see whether or not all those variables which had statistically significant association with the outcome variable in bivariate analysis, maintained their association in the multivariate logistic regression. Adjusted odds ratio with 95 % confidence interval (statistical significance was declared at *P* < 0.05) was used to show the significance of the association. The results were presented using absolute numbers, proportions, medians, mean, standard deviation, odds ratio and confidence intervals. Tables, figures and graphs were produced.

### Ethical consideration

The study was conducted after getting approval of the proposal from ethical review committee of Hawassa University. Written consent was obtained from Gurage Zone Health Department. Involvement into the study was on the basis of an informed consent that was obtained from each respondent, and for those age less than 18 the consent was obtained from the guardian or parents accordingly. Confidentiality was assured by avoiding personal identifier of the data and the data was kept in a secure place by the principal investigator.

## Result

### Socio demographic characteristics

A total of 816 childbearing women who had at least 1 birth in the past 2 years prior to the survey were interviewed, out of 845 childbearing women included in the sample, giving a response rate of 96.6 %. Out of the respondents 659 (80.8 %) were rural residents, 314 (38.48 %) of them were in the age range of 30–35 years with mean age of 29 year (±4.9 years) and 807 (98.90 %) them belong to Gurage ethnic group. About 470 (57.60 %) respondents were unable to read and write, 802 (98.28 %) were married, and 608 (74.51 %) of the women were housewives by occupation. Regarding religion, 363 (44.49 %) of them were Muslim and 305 (37.38 %) were Orthodox religion followers (Table 1).

**Table 1:** Selected Socio – demographic characteristics of respondents (*N* = 816) in Cheha district, Ethiopia, December 2012 to January 2013

Variables	Urban (*n* = 157)	Rural (*n* = 659)	Total (*n* = 816)
	*N* (%)	*N* (%)	*N* (%)
Age of respondents at interview			
15–19	2 (1.27 %)	11 (1.67 %)	13 (1.5 %)
20–24	36 (22.93 %)	76 (11.53 %)	112 (13.73 %)
25–29	70 (44.59 %)	231 (35.05 %)	301 (36.89 %)
30–35	40 (25.48 %)	274 (41.58 %)	314 (38.48 %)
35+	9 (5.73 %)	67 (10.17 %)	76 (9.31 %)
Ethnicity of respondents			
Gurage	150 (95.54 %)	657 (99.70 %)	807 (98.9 %)
Others	7 (4.46 %)	2 (0.3 %)	9 (1.1 %)
Respondents educational status			
Unable to read and write (Illiterate)	36 (22.93 %)	434 (65.86 %)	470 (57.6 %)
Only able to read and write	1 (0.64 %)	1 (0.15 %)	2 (0.25 %)
Elementary (Primary)	71 (45.22 %)	204 (30.96 %)	275 (33.7 %)
Secondary	35 (22.29 %)	13 (1.97 %)	48 (5.88 %)
Technical/Vocational +	14 (8.92 %)	7 (1.06 %)	21 (2.58 %)
Respondents marital status			
Currently married	154 (98.09 %)	648 (98.33 %)	802 (98.28 %)
Widowed	1 (0.64 %)	4 (0.61 %)	5 (0.61 %)
Divorced	2 (1.27 %)		2 (0.25 %)
Separated		3 (0.46 %)	3 (0.37 %)
Never married		4 (0.61 %)	4 (0.49 %)
Respondents occupation			
Housewife	70 (44.59 %)	538 (81.64 %)	608 (74.51 %)
Farmer	1 (0.64 %)	8 (1.21 %)	9 (1.10 %)
Government employee	19 (12.10 %)	7 (1.06 %)	26 (3.19 %)
Merchant	46 (29.30 %)	92 (13.96 %)	138 (16.9 %)
Other	21 (13.37 %)	14 (2.13 %)	35 (4.3 %)
Respondents religion			
Orthodox	55 (35.03 %)	250 (37.94 %)	305 (37.38 %)
Catholic	21 (13.38 %)	33 (5.01 %)	54 (6.62 %)
Protestant	12 (7.64 %)	82 (12.44 %)	94 (11.52 %)
Muslim	69 (43.95 %)	294 (44.61 %)	363 (44.49 %)
Respondent’s husband education (778)			
Unable to read and write (Illiterate)	7 (4.46 %)	115 (18.5 %)	122 (15.7 %)
Only able to read and write	3 (1.91 %)	9 (1.5 %)	12 (1.5 %)
Elementary (Primary)	78 (49.68 %)	384 (61.8 %)	462 (59.4 %)
Secondary	30 (19.11 %)	91 (14.7 %)	121 (15.6 %)
Technical/Vocational +	39 (24.84)	22 (3.5 %)	61 (7.8 %)
Income/month in Birr (*n* = 803)			
<450	13 (8.28 %)	134 (20.74 %)	147 (18.31 %)
450–799	51 (32.48 %)	357 (55.26 %)	408 (50.81 %)
800 or above	93 (59.24 %)	155 (23.99 %)	248 (30.88 %)
Respondent’s husband occupation (*n* = 812)			
Farmer	13 (8.3 %)	481 (73.4 %)	494 (60.8 %)
Government employee	57 (36.3 %)	38 (5.8 %)	95 (11.7 %)
Merchant	37 (23.6 %)	74 (11.3 %)	111 (13.7 %)
Other	50 (31.6 %)	62 (9.5 %)	112 (13.8 %)

### Obstetric, delivery and ANC characteristics

The majority of respondents (69 %) were married for the first time at the age of 18 years and above, and the mean age at first marriage was 18.8 year (±2.7) and 380 (46.57 %) of respondents had their first pregnancy below the age of 20 years with mean age at first pregnancy being 20.1 year (±2.9). Regarding gravidity, 293 (44.46 %) of rural women and 40 (25.48 %) of urban women had history of 5 and above pregnancies with mean gravidity for both urban and rural women being 4 (±2). About 281 (42.6 %) of rural respondents and 34 (10.8 %) of urban women had given 5 and above births in their previous reproductive life time with mean parity for both urban and rural mothers being 3.9 (±1.95). Out of the respondents 103 (12.62 %) of them encountered at least one abortion in their life time. Concerning ANC visit, 578 (87.71 %) of rural and 156 (99.36 %) of urban respondents had at least one prenatal visit while being pregnant to their last child (Table 2).

**Table 2:** Selected Obstetric and delivery characteristics of the respondents in Cheha District, Ethiopia, December 2012 to January 2013

Variables	Urban (*n* = 157)	Rural (*n* = 659)	Total (*n* = 816)
	*N* (%)	*N* (%)	*N* (%)
Mothers age at first marriage			
<18 years	39 (24.84 %)	214 (32.47 %)	253 (31 %)
18 year and above	118 (75.16 %)	445 (67.53 %)	563 (69 %)
Mothers age at first pregnancy			
<20 years	67 (42.68 %)	313 (47.50 %)	380 (46.57 %)
20 years and above	90 (57.32 %)	346 (52.50 %)	436 (53.43 %)
Gravidity			
1	30 (19.11 %)	55 (8.35 %)	85 (10.42 %)
2–4	87 (55.41 %)	311 (47.19 %)	398 (48.77 %)
≥5	40 (25.48 %)	293 (44.46 %)	333 (40.81 %)
Parity			
1	31 (19.7 %)	57 (8.7 %)	88 (10.8 %)
2–4	92 (58.6 %)	321 (48.7 %)	413 (50.6 %)
≥5	34 (21.7 %)	281 (42.6 %)	315 (38.6 %)
Had abortion in life time			
Yes	20 (12.74 %)	83 (12.59 %)	103 (12.62 %)
No	137 (87.26 %)	576 (87.41 %)	713 (87.38 %)
Use of ANC service (*n* = 734)			
Yes	156 (99.36 %)	578 (87.71 %)	734 (89.95 %)
No	1 (0.64 %)	81 (12.29 %)	82 (10.05 %)
Number of ANC visits (*n* = 734)			
<4×	27 (17.31 %)	157 (27.16 %)	184 (25.07 %)
≥4×	129 (82.69 %)	414 (71.63 %)	543 (73.98 %)
I don’t Know		7 (1.21 %)	7 (0.95 %)
First time received ANC (*n* = 730)			
4^th^ month or less	62 (28.6 %)	155 (71.4 %)	217 (29.7 %)
Above 4^th^ month	94 (18.3 %)	419 (81.7 %)	513 (70.3 %)
Place of delivery of the 2^nd^ youngest child (*n* = 728)			
Home	41 (32.5 %)	510 (84.7 %)	551 (75.7 %)
Health Institution	85 (67.5 %)	92 (15.3 %)	177 (24.3 %)

Among respondents who had ANC visit, 543 (73.98 %) of them visited health institutions 4 times and above for ANC service, and information (counseling) about where to give birth was given for 565 (77 %) of them. About 380 (52.49 %) women, out of those women who responded for the question, reported that they were informed about pregnancy complications at ANC clinic (Table 2).

**Fig. 1:**
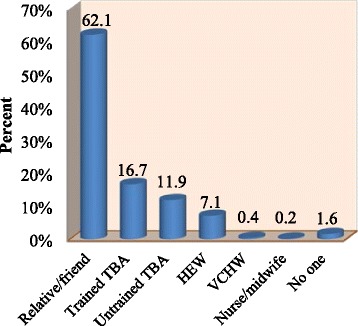
Attendants of Home Delivery in Cheha District, Ethiopia, December 2012 to January 2013

Regarding place of delivery and attendants of the birth of the last under 2 year child, 253 (31.0 %) of all respondents gave birth to the youngest child in health institution by skilled birth attendants, and when we see across residential area 128 (19.4 %) of rural and 125 (79.6 %) of urban women gave birth in health institutions by skilled birth attendants. Majority of home deliveries (62.1 %) were assisted by relative and/or friends (Fig. 1).

**Fig. 2:**
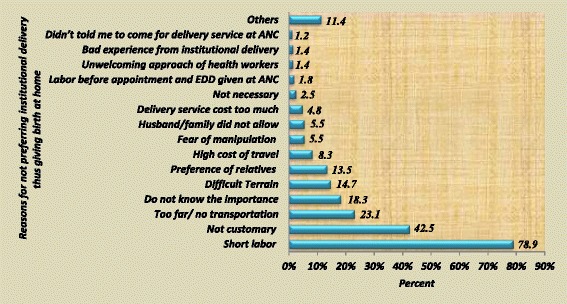
Reasons given by women for not preferring institutional delivery thus giving birth at home in Cheha District, Ethiopia, December 2012 to January 2013 (more than one answer is possible)

Respondents who gave birth to their last under 2 year child at home were asked about their reasons for not preferring utilization of health institutions for delivery service, and 444 (78.9%) of them mentioned short labor as one of their reason, 239 (42.5%) of them reported that it is not customary to give birth at health institution, 130 (23.1%) of them reported that they gave birth at home because the health institution is too far and/or no transportation among others (Fig. 2).

Out of 563 women who gave birth at home, 559 of them responded for complications they encountered while giving birth at home, and 127 (22.72 %) of them reported one or more types of complications. Regarding types of complication, 50 (39.68 %) women reported excessive bleeding, 43 (33.86 %) reported retained placenta, 38 (30.16 %) encountered prolonged labor and 26 (20.63 %) reported high fever.

Among women who gave birth at health institution, 79.4 % women had preplanned to give birth at health facility, 20.2 % were referred to health facility by community due to problem they encountered during labor and 0.4 % reported that it happened by chance while in health facility for other purposes.

### Factors associated with safe delivery service utilization

Bivariate analysis was done to see the association of socio-demographic factors, access to health service, health care behavior, cultural characteristics, obstetric factors, knowledge and attitude, to the outcome of interest which is institutional delivery (that is attended by skilled birth attendants). All those variables which were found to be associated with outcome variable (*p* value less than 0.05) and the well known confounders were included in the multivariate logistic regression model and the following variables maintained their association:

Place of residence was found to be one of the most important predictor of institutional delivery. Mothers residing in urban areas were 3.3 times more likely to give birth at health facility than mothers residing in rural areas (OR = 3.26, 95 % CI: 1.33, 7.97). Traveling time (distance) needed to reach to the nearby health institution which can provide safe delivery service was very important factor that influences institutional delivery. Women who should travel more than 60 min were less likely to deliver at health facility than those women who travels less than 30 min to the nearest health facility that can provide safe delivery service (OR = 0.22, 95 % CI: 0.09, 0.55). Husband attitude was another very important factor. Women having a husband with negative attitude towards institutional delivery were less likely to give birth at health facility than those women having a husband with positive attitude (OR = 0.19, 95 % CI: 0.04, 0.80). Women’s ability to afford for the whole process of getting delivery service at health institution was significantly associated with institutional delivery. Women who can afford to pay for the whole process of going from home to health facility and for the fee that needs to paid at health institution for delivery service were 2.5 times more likely to gave birth at health facility than their counterparts (OR = 2.48, 95 % CI: 1.11, 5.53). Counseling service, during last child pregnancy, at ANC clinic about where to give birth had strong associations with institutional delivery. Women who were not counseled at ANC about where to give birth were less likely to give birth at health facility than their counterparts (OR = 0.37, 95 % CI: 0.17, 0.84). Place of birth of 2^nd^ youngest child was found to be another important predictor for institutional delivery. Women who gave birth to their 2^nd^ youngest child at health institution were 7 times more likely to give birth to their youngest child at health facility than those women who gave birth to their 2^nd^ youngest child at home (OR = 7.23, 95 % CI: 4.14, 12.62) (Table 3).

**Table 3:** Factors Associated with Institutional Delivery Service Utilization among Childbearing Mothers in Cheha District, Ethiopia, December 2012 to January 2013

Variable	Institutional delivery attended by skilled birth attendants		Crude OR (95 % CI)	Adjusted OR (95 % CI)
	Yes	No		
	*N* (%)	*N* (%)		
Place of residence				
Urban	125 (79.62 %)	32 (20.38 %)	**16.21 (10.50, 25.00)**	**3.26 (1.33, 7.97)**
Rural	128 (19.42 %)	531 (80.58 %)	**1.00**	**1.00**
Husbands attitude towards institutional delivery				
Negative	6 (4.58 %)	125 (95.42 %)	**0.09 (0.04, 0.20)**	**0.19 (0.04, 0.80)**
Positive	243 (35.68 %)	438 (64.32 %)	**1.00**	**1.00**
Traveling time needed to reach to health facility that provide safe delivery service				
>60 min	39 (11.82 %)	291 (88.18 %)	**0.04 (0.03, 0.08)**	**0.22 (0.09, 0.55)**
30–60 min	129 (34.58 %)	244 (65.42 %)	**0.17 (0.11, 0.28)**	**0.42 (0.18, 0.95)**
<30 min	85 (75.22 %)	28 (24.78 %)	**1.00**	**1.00**
Able to afford for the whole process of getting delivery service at health facility				
Yes	236 (34.40 %)	450 (65.60 %)	**3.46 (2.03, 5.89)**	**2.48 (1.11, 5.53)**
No	17 (13.18 %)	112 (86.82 %)	**1.00**	**1.00**
Knowledge on danger signs and delivery service				
Not knowledgeable	118 (21.03 %)	443 (78.97 %)	**0.24 (0.17, 0.33)**	0.68 (0.37, 1.24)
Knowledgeable	135 (52.94 %)	120 (47.06 %)	**1.00**	1.00
Attitude towards institutional delivery				
Unfavourable	3 (9.68 %)	28 (90.32 %)	**0.23 (0.07, 0.76)**	0.17 (0.02, 1.14)
Favourable	250 (31.85 %)	535 (68.15 %)	**1.00**	1.00
Use of ANC service				
No	2 (2.44 %)	80 (97.56 %)	**0.05 (0.01, 0.20)**	0.27 (0.06, 1.17)
Yes	251 (34.20 %)	483 (65.80 %)	**1.00**	1.00
Counseling given at ANC clinic about where to give birth				
No	13 (11.61 %)	99 (88.39 %)	**0.23 (0.13, 0.43)**	**0.37 (0.17, 0.84)**
Yes	163 (35.98 %)	290 (64.02 %)	**1.00**	**1.00**
Place of birth of 2^nd^ youngest child				
Health institution	129 (72.9 %)	48 (27.1 %)	**21.20 (13.87, 32.38)**	**7.23 (4.14, 12.62)**
Home	62 (11.3 %)	489 (88.7 %)	**1.00**	**1.00**

Adjusted for Socio-demographic variables including access related factors, health care behaviours and cultural factors; obstetric variables including characteristics of ANC service; knowledge and attitude of respondents. The results that are written in bold numbers indicate the results of those variables that show statistically significant association with the outcome variable in the Crude OR and Adjusted OR analysis

## Discussion

This community based cross sectional study attempted to measure magnitude of institutional delivery and to identify factors associated with institutional delivery service utilization among childbearing women in both urban and rural areas of Cheha District, Ethiopia. In this study, the major predictors of using health facility for delivery service were found to be place of residence, ability to afford for the whole process of getting delivery service at health facility including transportation cost and delivery service fee at health facility, husbands attitude towards institutional delivery, place of birth of the second youngest child, provision of counseling service at ANC clinic about where to give birth during ANC visit for the last child pregnancy, and traveling time (distance) needed to reach to nearest health facility where women can find safe delivery service.

The study revealed that the prevalence of institutional delivery attended by skilled providers, which can be justified as the best bet to bring down high rates of maternal mortality [4], to be 31 %. The remaining 69 % women gave birth at home, which exposes many of them to complications among which are excessive bleeding, retained placenta, prolonged labor and high fever. The majority (62.1 %) of home deliveries were attended by relatives and/or friends who are not expected to handle these complications. It is well established fact that most maternal deaths happen due to complications that occur during labor, delivery, and postpartum period, particularly hemorrhage being the leading cause of maternal death [3]. The low prevalence of institutional delivery in the district might be leading to a high number of maternal and neonatal morbidity and mortality. This calls for an accelerated, integrated and appropriate interventions to be carried out by all stakeholders to increase institutional delivery which is attended by skilled providers. Unless appropriate actions are taken on time it might be difficult to achieve MDG 5.

Our finding is similar with a study conducted in Nepal where 31 % of women had delivered their last baby in hospital but lower than the finding of institutional delivery service coverage in other studies such as India (63 %), Chongwe district in Zambia (42.8 %) and Bahi district in Tanzania (54 %) [26–29]. This figure is also lower than the prevalence of skilled birth attendance estimated to occur in Sub Saharan Africa and even lower than the prevalence estimated for Eastern Africa where the lowest (33.7 %) skilled delivery attendance is registered [12, 13]. The relatively better coverage in utilization of health facility for delivery service in the aforementioned countries as compared to our findings might be due to the socioeconomic including infrastructure and the cultural differences that might exist across these countries. However, it is better than the findings of health facility delivery coverage in different parts of Ethiopia [19–22, 30]. This finding is also higher than the 2011 EDHS report of both institutional delivery and skilled birth attendance coverage of 9.9 and 10 % respectively [25]. The relatively better institutional delivery service coverage we found in the district as compared to the other figures in the nation might be due to the current better health facility distribution in the district. Besides expansion of health facilities by the government there are 1 clinic, 1 health center and 1 hospital run by faith based organizations (FBO’s) in rural parts of the district where the government health centers are not found. The awareness creation by a dedicated health extension workers might have a role as well. Nevertheless, a lot remains to be done to improve utilization of health facility (that can provide delivery service by skilled providers) for delivery service by childbearing mothers in the district.

In this study one of the most important predictor for institutional delivery was found to be women’s place of residence. Women who are living in urban areas had strong odds of using health facility for delivery service than their rural counterparts. This finding is consistent with different studies conducted in India, Sub-Saharan Africa and Ethiopia [19–22, 31–33]. This may be due to the fact that the physical accessibility of health facilities in urban areas with lesser transportation cost could encourages mothers to give birth at health facility in addition to the better access to information of the urban community with regards to the benefit of delivering at health facility. On the contrary, in rural areas, the poor physical access to health facilities which provide safe delivery service, the difficult terrains with lack of roads, lack of transportation system and high cost of travel might hinder women to deliver at health facility. In this study, most women residing in rural areas who gave birth at home also reported the majority of the aforementioned reasons as their perceived problems for not giving birth at health institution. This suggests that accessibility issue, one of the factors that contribute to phase 2 delays of health service use [16], is influencing utilization of health facility that can provide safe delivery service - which is one of the most important factors that helps in reducing maternal mortality [3, 34, 35].

Husband’s attitude towards institutional delivery was the other factor that is found to be influencing utilization of health facility for delivery service. Women having a husband with negative attitude towards institutional delivery were less likely to give birth at health facility than women whose husbands have positive attitude towards this service. This finding is in line with studies conducted in Arsi, Ethiopia [19]. Husbands having negative attitude towards institutional delivery might not support their wife to go to health facility for delivery service as they are usually the decision maker in major events including the expenses in the household. Besides, most women in developing countries do not have their own income. This finding indicates that the support from husband is very helpful for the women to use health facility for delivery service.

The study found that traveling time (distance) needed to reach to the nearby health institution, which can give safe delivery service, as one of the most important predictor of utilization of health facility for delivery service. Women who have to travel more than one hour were less likely to give birth at health facility than women who travels less than one hour. This finding suggests that if health institution that can provide safe delivery service is physically accessible, it will boost the likelihood of using the health facility for delivery service. When the health institution is found more closer the cost needed to get there will also be reduced, the difficult terrain that a women needs to travel will decrease and she might get better social support because people do not need to travel a long distance escorting and/or carrying her. This finding of the study is very consistent with other studies conducted in Afghanistan, Indonesia, Tanzania and Ethiopia [24, 29, 36, 37].

Ability to afford for the whole process that takes to reach to health facility and for the delivery service fee that is asked to be paid at health institution was important factor affecting institutional delivery. Women who can afford for the whole process that takes to reach to health facility and able to pay for delivery service had greater odds of giving birth at health institution than their counterparts. This might be due to the fact that some of the health institutions were asking some amount of money for the service. Besides money is needed to pay either for transportation and/or to buy food for men who carries the women in labor, by locally prepared strature (bed), from home to health institution. This indicates that having sufficient amount of cash on hand increases the likelihood of the women to give birth at health institution. This is in line with other studies conducted in Indonesia, India and Ethiopia [10, 14, 16, 24, 37].

Provision of information (counseling) for women about place of delivery during ANC visit while she was pregnant the last child was the other important predictor for using health facility for delivery service. Women who were not counseled about where to give birth during their ANC visit were less likely to give birth at health facility than their counterparts. This finding suggests that it is not only going to health facility for ANC service which increases utilization of health facility for delivery service, like the other study finding in Ethiopia [19–22], it is rather the provision of counseling service during ANC visit about where to give birth that predicts the service utilization.

Another very important predictor for institutional delivery was found to be place of birth of the 2^nd^ youngest child. Women who gave birth to their 2^nd^ youngest child at health institutions had strong odds of using health facility for delivery of their last child than women who gave birth their 2^nd^ youngest child at home. This finding indicates that women might have the tendency to continue giving birth to their next child in health institution once they had experience of giving birth at health institution. This tendency could be due to an experience of better outcome and getting information about the benefits of institutional delivery. It also indicates that there is a tendency to continue giving birth at home once the mother had previously given birth at home. And if these women continued to give birth at home, deleterious complications might arise leading to high maternal morbidity and mortality [3]. This finding is in agreement with the studies conducted in Zambia and elsewhere [14, 28].

### Limitations of the study

The result of this study should be interpreted carefully as it might be difficult to establish cause- effect relationship due to the cross sectional nature of the study. Even if the training of data collectors as well as supervisors with the supply of questionnaire for interviewing and the memorable nature of most of the events can have a significant impact in minimizing biases, it might still be difficult to exclude recall bias as some participants were expected to remember events that happened up to 2 years back.

The relatively sensible proportion of urban and rural participants from reasonable number of rural and urban kebeles with larger sample size, and the design of the study being community based study which used probability sampling technique that helps to minimize selection bias thus can be generalized for urban and rural area, are some of the strengths that can give adequate power for the study regardless of its limitations.

## Conclusion and Recommendation

This study demonstrates that institutional delivery service utilization in the district is low. There is great disparity between urban and rural areas in using health facility for delivery service. Besides place of residence, the most important factors associated with institutional delivery were found to be traveling time that takes to reach to health institution, ability to afford for the whole process (transportation and delivery service fee) of getting delivery service at health facility, husband’s attitude towards institutional delivery, counseling during ANC visit about where to give birth and place of birth of the 2^nd^ youngest child. If most mothers carry on delivering at home they will continue to encounter birth related complications and the high maternal morbidity and mortality will persist. Hence all stakeholders including policy makers, planners and health service managers should distinguish factors impeding institutional delivery and work strongly to curb the situation. They should give attention to improve accessibility of health institution that can provide safe delivery service. Husbands and the community at large should get health education and BCC services to promote positive behavior towards institutional delivery. Health professionals should counsel and encourage all pregnant women who visits ANC clinic to give birth at health institutions where safe delivery service is available. In our study ANC attendance didn’t show impact on institutional delivery, rather it is the counseling service provided during ANC visit about where to give birth that had an impact on the service utilization. This calls for exploration of quality of ANC service by all stakeholders and applying appropriate intervention to improve the service. It is also absolutely imperative to work on schemes which improve economic status of women and to boost women’s decision power over income to enhance institutional delivery service utilization.
